# Diet-Modulated Lipoprotein Metabolism and Vascular Inflammation Evaluated by ^18^F-fluorodeoxyglucose Positron Emission Tomography

**DOI:** 10.3390/nu10101382

**Published:** 2018-09-28

**Authors:** You-Bin Lee, Kyung Mook Choi

**Affiliations:** Division of Endocrinology and Metabolism, Department of Internal Medicine, Korea University Guro Hospital, Korea University College of Medicine, 148 Gurodong-ro, Guro-gu, Seoul 08308, Korea; yb.snowyday1224@gmail.com

**Keywords:** positron emission tomography, atherosclerosis, inflammation, lipoprotein, diet

## Abstract

Vascular inflammation plays a central role in atherosclerosis, from initiation and progression to acute thrombotic complications. Modified low-density lipoproteins (LDLs) and apoB-containing particles stimulate plaque inflammation by interacting with macrophages. Loss of function of high-density lipoprotein (HDL) for preventing LDL particles from oxidative modification in dyslipidemic states may amplify modified LDL actions, accelerating plaque inflammation. Diets are one of the most important factors that can affect these processes of lipoprotein oxidation and vascular inflammation. Recently, ^18^F-fluorodeoxyglucose (FDG) positron emission tomography (PET) has emerged as a reliable noninvasive imaging modality for identifying and quantifying vascular inflammation within atherosclerotic lesions based on the high glycolytic activity of macrophages infiltrating active atherosclerotic plaques. Vascular inflammation evaluated by FDG PET has been positively related to metabolic syndrome components and traditional risk factors of cardiovascular disease, including high-sensitivity C-reactive protein, body mass index, and insulin resistance. A positive association of vascular inflammation with endothelial dysfunction, resistin levels, pericardial adipose tissue, and visceral fat area has also been reported. In contrast, HDL cholesterol and adiponectin have been inversely related to vascular inflammation detected by FDG PET. Because of its reproducibility, serial FDG PET shows potential for tracking the effects of dietary interventions and other systemic and local antiatherosclerotic therapies for plaque inflammation.

## 1. Introduction

Atherosclerotic cardiovascular disease (ASCVD), manifested as various forms of fatal diseases including myocardial infarction, ischemic stroke, and peripheral artery occlusive disease, is the leading cause of morbidity and mortality [1]. Therefore, developing methods for identifying and monitoring the atherosclerotic process in early phases may greatly impact public health. Accumulating data from extensive research indicate that modified lipoproteins and subsequent vascular inflammation are key factors in the atherosclerotic process [2,3]. The oxidation of lipoproteins and vascular inflammation may be significantly influenced by dietary patterns [4,5], suggesting that this may be a link between diet and ASCVD [5,6]. Recently, ^18^F-fluorodeoxyglucose (FDG) positron emission tomography (PET) has attracted attention as an imaging modality for detecting vascular inflammation in atherosclerotic plaques early in relation to these key factors of arteriosclerosis. The main aim of this review is to describe the interactions between lipoprotein and macrophages leading to vascular inflammation during atherosclerosis, effects of diets during this process of inflammation, and evaluation of this vascular inflammation by ^18^F- FDG PET. 

## 2. Atherosclerosis, an Inflammatory Disease

Inflammation is known to play a key role in the development and progression of atherosclerosis [3], and atherosclerosis is currently considered as a low-grade vascular inflammation [3]. Inflammation contributes to all stages of atherosclerotic cardiovascular disease, from the earliest initiation and evolution of atherosclerosis to acute thrombotic complications [3]. Inflammation not only induces thrombus formation but also inhibits endogenous fibrinolysis such that once formed, the thrombus is firmly maintained [3]. Acute cardiovascular events are thought to be affected mainly by the inflammatory status and constitution of atherosclerotic plaques rather than the extent of vascular stenosis [7]. 

**Figure 1:**
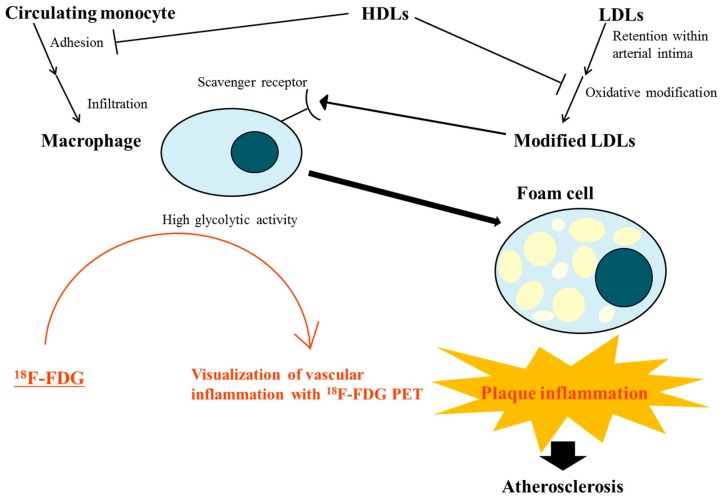
Interplay of lipoproteins and macrophages linked to plaque inflammation during atherosclerotic process and evaluation of this vascular inflammation by ^18^F-fluorodeoxyglucose (FDG) positron emission tomography (PET). Modified low-density lipoproteins (LDLs) trigger plaque inflammation by interacting with macrophages. The macrophages ingest modified LDL through scavenger receptors and evolve into foam cells, which play a key role in the development and progression of atherosclerosis. High-density lipoproteins (HDLs) protect LDLs from oxidative modification and inhibit monocyte adhesion to the endothelium by decreasing the expression of adhesion molecules in endothelial cells. Loss of this protective function of HDL in dyslipidemic states may accelerate plaque inflammation, amplifying the role of modified LDLs. The high glycolytic activity of infiltrated macrophages enables visualization of vascular inflammation in atherosclerotic lesions by ^18^F-FDG PET.

Activated macrophages are among the major contributors to increased susceptibility of plaque rupture and promoting thrombus formation [3]. Macrophages ingest lipids through the expression of scavenger receptors for modified lipoproteins and develop into foam cells [3] (Figure 1). Physical disruptions of atherosclerotic plaques cause most coronary arterial thrombi that lead to fatal acute myocardial infarction [3]. The activated macrophages in atheroma can secrete proteolytic enzymes, which cause thinning and weakening of the protective fibrous cap of the plaque by degrading collagen in the cap [3]. This makes the plaque more vulnerable to rupture [3]. Activated macrophages can also express tissue factors, which act as the main procoagulants and induce thrombosis in plaques [3].

Elevated circulating inflammatory markers, particularly C-reactive protein (CRP), can predict the risk of atherosclerotic cardiovascular events [3,8] and poor outcomes in acute coronary syndromes [3]. Increasing evidences from population-based studies support that low-grade chronic inflammation, represented by increased levels of CRP, defines the future risk of atherosclerotic complications [3,8]. Therefore, CRP testing may have an important adjunctive role in estimating future cardiovascular risk in addition to the use of traditional risk factors [3,8], and combining CRP and lipid testing to improve cardiovascular risk prediction may have clinical utility [3,8]. 

Data from experimental and clinical studies indicate that statins not only lower low-density lipoproteins (LDLs) but also reduce plaque inflammation and affect plaque stability [3,9]. Macrophage contents in experimental atherosclerotic plaques can be reduced by pravastatin [3,10,11]. Simvastatin, fluvastatin, and atorvastatin are known to inhibit the expression of tissue factor and matrix metalloproteinases in vivo as well as in vitro [12,13] and appear to attenuate intimal inflammation [14]. Statins may also suppress the expression of adhesion molecules, thus reducing the attachment and adhesion of monocytes to the vascular endothelium [15]. The Cholesterol and Recurrent Events (CARE) investigators reported that CRP levels were lowered by pravastatin independently of the effect of pravastatin on high-density lipoprotein (HDL) or LDL cholesterol [16]. Reduced CRP levels by statins were also demonstrated in clinical studies of lovastatin, simvastatin, and atorvastatin [17,18,19]. 

CRP testing may be useful for targeted statin therapy, particularly for the primary prevention of cardiovascular events [3]. In the Justification for the Use of Statins in Prevention: an Intervention Trial Evaluating Rosuvastatin (JUPITER) study, incident cardiovascular disease (CVD) was reduced by 44% by rosuvastatin therapy in apparently healthy persons without hyperlipidemia but with increased high-sensitivity CRP (hsCRP) levels [20]. According to the CARE investigators, the proportion of recurrent coronary events prevented by pravastatin was greater among those with evidence of inflammation, which was defined by the CRP and serum amyloid A levels, compared to those without evidence of inflammation [16,21]. The Air Force/Texas Coronary Atherosclerosis Prevention Study (AFCAPS/TexCAPS) CRP substudy suggested that CRP screening can be conducted to improve the targeting of statin therapy adjunctively with lipid testing [3,19].

## 3. Effects of Lipoproteins on Vascular Inflammation

Modified LDL and other apoB-containing particles represent key stimulators of plaque inflammation through interplay with macrophages [2] (Figure 1). The endothelial layers are penetrated by atherogenic lipoproteins at sites of activation, such as branch points of arteries [2,22], and all forms of apoB-containing particles, including LDL, very low-density lipoprotein (VLDL), VLDL remnants, and lipoprotein(a), have the potential for retention within the arterial intima [2,22]. Once retained, LDL may undergo modification by aggregation, lipolysis, oxidation, or proteolysis [2]. According to the oxidation hypothesis of atherosclerosis, this oxidative modification of lipoproteins, primarily LDL, in the vascular wall constitutes a critical event in atherogenesis [2,23]. Modified LDL, frequently including oxidized lipids, chronically stimulates innate and adaptive immune reactions [2,3], resulting in activation of endothelial cells and smooth muscle cells [2,24]. Accordingly, these activated cells express adhesion molecules, chemoattractants, and growth factors to attract circulating monocytes [2,24]. Subsequently, circulating monocytes migrate into the intima and differentiate into macrophages or dendritic cells [2,24]. Intimal monocyte-derived macrophages engulf modified LDLs through their scavenger receptor or other pathways and convert into foam cells packed with droplets of lipoprotein-derived cholesteryl esters [2,25]. These foam cells, exhibiting a proinflammatory phenotype, represent an essential element in the early fatty streak change and play a central role in lesion progression [2]. In these series of processes, it is noteworthy that it is the modified LDL that constitutes the primary driver for foam cell formation [2]. The binding of modified LDL to pattern recognition receptors on macrophages is now thought to be the primary trigger in plaque inflammation [2,22,24,25,26,27].

Loss of the function of HDL to protect LDL particles from oxidative modification in dyslipidemic states, including hypertriglyceridemia, may amplify the role of modified LDL and accelerate intraplaque inflammation [2] (Figure 1). HDL particles exert protective activities against atherosclerosis by effluxing cellular cholesterol, attenuating vascular inflammation, decreasing intracellular oxidative stress and platelet activation, relieving vascular constriction, and sustaining glucose homeostasis [2]. Decreased plasma HDL levels and/or HDL particle numbers may impair the clearance of cholesterol from arterial wall cells, primarily from macrophages and macrophage-derived foam cells [2,3]. HDL particles inhibit chronic inflammation in the arterial wall, which is central in atherosclerosis, through multiple anti-inflammatory actions [2]. HDLs decrease the expression of adhesion molecules in endothelial cells and subsequently suppress monocyte adhesion to the endothelium [2,28,29,30,31,32]. They also reduce monocyte activation through nuclear factor kappa B (NF-κB) and peroxisome proliferator-activated receptor gamma (PPAR-gamma)-dependent pathways [2,33,34,35]. Additionally, apoptosis of both macrophages and endothelial cells by free cholesterol or oxidized LDL loading may be protected by HDL particles [2,36]. Moreover, HDL particles decrease reactive oxygen species generation and intracellular oxidative stress [37,38,39,40,41]. Antioxidant enzymes, including platelet-activating factor acetyl hydrolase and paraoxonase, can also be transported by HDL particles to degrade oxidized lipids and alleviate their proinflammatory effects [3]. Loss of the antioxidative action of HDL in dyslipidemic states can aggravate the build-up of LDL-derived oxidized phospholipids with proinflammatory effects and amplify inflammation in the arterial intima [2,42]. 

## 4. Impact of Diets on Inflammation and Lipoprotein Oxidation Modulating Atherosclerotic Process

Effect of diets on LDL oxidation and inflammation has been consistently reported [4,5] and suggested as a possible link between diet and CVD [5,6]. When Mata et al. [4] examined plasma LDL and HDL cholesterol levels, in vitro LDL oxidation, and monocyte adhesion to cultured human endothelial cells in 42 healthy individuals by subjecting them to four consecutive diets with different fat content of saturated fatty acids, monounsaturated fatty acids, and polyunsaturated fatty acids (n-6) and (n-3), a monounsaturated fatty acid-rich diet was associated with more favorable plasma lipid concentrations compared with a saturated fatty acid-rich diet. In addition, this diet was demonstrated to induce a higher resistance of LDL to copper-induced oxidation and a lower monocyte adhesion rate to endothelial cells than other diets with various fatty acid contents [4]. Increased substrate concentration in postprandial states, including both postprandial hypertriglyceridemia and hyperglycemia, are known to generate endothelial dysfunction, one of the earliest indicators of atherosclerosis [5]. The increased level of triglyceride following a high-fat (saturated) diet was inversely related to endothelial function, particularly when accompanied by postprandial hyperglycemia when Nappo et al. [43] explored the effect of various meals with identical calories on endothelial function in participants with and without type 2 diabetes. This impact of postprandial hypertriglyceridemia and hyperglycemia on endothelial function may be mediated by increased oxidative stress accompanied by meal intake, which in turn leads to elevated circulating biomarkers of inflammation and adhesion [5,44]. Inactivation of nitric oxide by increased superoxide production and peroxynitrite generations through combination of superoxide with nitric oxide are considered to be involved in these series of processes [5]. Lipid peroxidation is initiated by the peroxynitrite, which constitutes a potent and long-lasting oxidant with cytotoxic properties [5].

Increasing evidences indicate that induction or alleviation of a proinflammatory condition and subsequent alteration in endothelial function by dietary patterns may be one mechanism that CVD risks are affected by diets [5]. Ingestion of certain macronutrients may induce oxidative stress and inflammatory milieu [5]. Glucose intake is associated with increased superoxide production in leukocytes and mononuclear cells and activation of proinflammatory transcription factors including NF-κB in normal subjects [5]. A high-fat meal promotes endothelial activation, manifested by increased adhesion molecule levels [5,43], and elevation in proinflammatory cytokine interleukin-18 that can weaken plaque stability [5,45]. In contrast, increased consumption of fruits, vegetables, and fibers that are abundant in natural antioxidants largely reduce the adverse effect of high-fat-meal-induced oxidative stress [5]. In addition to this dietary strategy, increasing omega-3 fatty acid and decreasing trans and saturated fatty acids consumption, which are known to prevent coronary heart disease [46], have been related to decreased inflammatory status [5]. Higher trans-fatty acid consumption was associated with increased CRP levels and markers of endothelial dysfunction [47], whereas low-cholesterol/low-saturated fat diets [48] and higher omega-3 fatty acid intake [49] were correlated with reduced plasma CRP concentrations. In a randomized trial with 180 patients with metabolic syndrome, a Mediterranean-style diet significantly reduced vascular inflammatory markers, including serum hsCRP and interleukin-18, and improved insulin resistance and endothelial function [50]. At two years of follow-up, a significant decrease in prevalence of metabolic syndrome was observed in the Mediterranean-style diet group compared to the control subjects [50]. Moreover, Zhong et al. [6] demonstrated that more proinflammatory diets, assessed by a novel dietary inflammatory index score, were independently associated with an increased risk of CVD, cardiovascular, and all-cause mortality in the general population.

Furthermore, some animal studies have tried to evaluate the effect of dietary modification on vascular inflammation and plaque contents of atherosclerotic lesions more directly. Verhamme et al. [51] reported that dietary cholesterol withdrawal induced reduction in LDL, oxidized LDL and CRP levels, restoration of endothelial function, and decrease in lipid, oxidized LDL and macrophage contents of atherosclerotic plaques in miniature pigs. Hartung et al. [52] used histologic and immunohistochemical analysis of the atherosclerotic lesions of rabbit models to show that dietary modification resolved macrophage infiltration, reduced apoptosis of macrophages, and increased smooth muscle cell content within the atherosclerotic plaques.

In clinical testing, there have been trials determining the long-term effect of dietary intervention on markers of vascular inflammation and plaque stability. A 12-month randomized trial with 164 individuals at high risk for CVD showed that enhanced Mediterranean diets attenuated the biomarkers related to atherosclerotic vascular inflammation and plaque vulnerability, including adhesion molecule expression on monocyte surface, CRP, interleukin-6, and endothelial adhesion molecules, compared to a low-fat diet [53]. According to the same group, Mediterranean diets not only improved lipid profiles, including total, LDL, and HDL cholesterol concentrations, but also reduced cellular and plasma inflammatory markers of atherogenesis, such as hsCRP, interleukin-6, and molecules associated with the attraction and adhesion of monocytes to vascular endothelium, compared to a low-fat diet at a longer-term follow-up of three and five years in a randomized controlled trial with high-risk adults without overt CVD [54].

## 5. Utility of ^18^F-fluorodeoxyglucose (FDG) Positron Emission Tomography (PET) for Identifying Vascular Inflammation

Recently, ^18^F-FDG PET has been established as one of the most useful imaging modalities for identifying and measuring inflamed vulnerable atherosclerotic plaques [55]. ^18^F-FDG PET is generally used in clinical fields to detect ^18^F-FDG uptake in cells with increased glucose metabolism [56], including inflammatory cells and tumor cells [57]. It has been reported that ^18^F-FDG accumulation is also increased by active atherosclerosis [56,58,59]. Inflammatory cell infiltration, particularly of macrophages, and subendothelial proliferation of smooth muscle cells and macrophages within atherosclerotic foci are considered to be highly associated with ^18^F-FDG uptake on PET images [60,61]. Visualization of vascular inflammation by ^18^F-FDG PET is attributed to the high glycolytic activity of macrophages within active atherosclerotic plaques [56,62] (Figure 1). Rudd et al. [63] reported that inflammatory plaques were identified by ^18^F-FDG PET in eight patients followed by carotid endarterectomy. Rogers et al. [64] described that ^18^F-FDG PET showed a higher uptake within the ascending aorta, left main coronary artery, and the culprit lesion in 10 patients with recent acute coronary syndrome compared to 15 individuals with stable angina. In the preclinical testing, Ogawa et al. [65] demonstrated that ^18^F-FDG accumulation in atherosclerotic lesions was ascribable to macrophages in a rabbit model of atherosclerosis. According to Tawakol et al. [66], ^18^F-FDG PET signals from carotid plaques were significantly correlated with macrophage staining of corresponding pathologic specimens isolated after endarterectomy. These studies indicate that ^18^F-FDG PET is a useful noninvasive tool for detecting and quantifying inflammation within atherosclerotic lesions, particularly macrophage-associated vascular inflammation [60,67].

Moreover, because of the high reproducibility of ^18^F-FDG uptake measurements [60,68,69], PET scanning may be useful for tracking the effects of atherosclerosis treatment [69]. Tahara et al. [60] reported good reproducibility and intraobserver and interobserver variability of less than 5% in standardized uptake value (SUV) measurements of plaque inflammation. The SUV is calculated as the decay-corrected tissue concentration of FDG divided by the administered dose per body weight [69]. The mean and maximum blood-normalized SUVs, known as the target-to-background ratio (TBR), are widely used to measure vascular ^18^F-FDG uptake [69]. Both the mean and maximum TBR have been reported to be equally reproducible [69]. Rudd et al. [69] suggested that systemic arterial therapies can be tracked by the mean TBR, whereas the maximum TBR can be used to monitor local, plaque-based therapy. In summary, serial ^18^F-FDG PET imaging may be helpful for monitoring the plaque burden and inflammatory activity [60] and used to examine responses in vascular inflammation to medical or local therapy because of its reproducibility [69].

## 6. Association of Vascular Inflammation Assessed by Positron Emission Tomography (PET) with Markers of Lipoprotein Metabolism and other Risk Factors for Atherosclerotic Cardiovascular Disease (ASCVD)

The association of vascular inflammation with markers of lipoprotein metabolism and other risk factors for ASCVD has been explored using ^18^F-FDG PET imaging in previous studies. A positive relationship between components of metabolic syndrome or traditional risk factors for ASCVD and vascular inflammation has been demonstrated [70]. Vascular inflammation quantified by FDG uptake in carotid atherosclerosis was positively related to waist circumference, hypertensive medication, and homeostasis model assessment of insulin resistance (HOMA-IR) and inversely associated with HDL cholesterol in 216 subjects who underwent cancer screening [70]. In the same study, age- and gender-adjusted FDG accumulation in carotid artery was increased in proportion to the satisfied number of metabolic syndrome components, suggesting an association between metabolic syndrome and vascular inflammation [70]. Additionally, hypercholesterolemia showed consistent positive correlations with FDG uptake in three different arteries, including the abdominal aorta, lilac, and proximal femoral arteries of 156 patients [61]. Studies confirmed a significant positive correlation of vascular inflammation with total cholesterol [56], blood pressure [71], body mass index (BMI) [71], waist circumference [70,71], waist-to-hip ratio [71], and insulin resistance [70]. A negative relationship between vascular ^18^F-FDG index and HDL cholesterol was consistently observed in other studies [56,70].

Inflammation within atherosclerotic plaques detected by ^18^F-FDG PET has been positively correlated with the circulating inflammatory marker hsCRP [70,71,72]. Vascular inflammation investigated by PET/computed tomography (CT) was reported to be increased in healthy individuals without hyperlipidemia but with high hsCRP values, suggesting that ^18^F-FDG uptake reflects early inflammatory changes in vascular walls in low-risk individuals [72]. In the same study, hsCRP and diastolic blood pressure were found to be independent determinants of vascular inflammation represented by maximal TBR [72]. Other studies also confirmed a significant positive relationship between hsCRP and vascular inflammation measured by FDG uptake [70,71,73].

Honda et al. confirmed that vascular inflammation in carotid arteries assessed by ^18^F-FDG PET was independently associated with endothelial dysfunction represented by a decreased percentage (%) flow-mediated dilation of the brachial arteries in 145 adults [74]. Endothelial dysfunction is one of the earliest steps in the atherosclerotic process and was reported to be correlated with several cardiovascular risk factors, including dyslipidemia, smoking status, obesity, insulin resistance, diabetes, hypertension, and CRP [74]. 

The association between vascular inflammation and serum adiponectin or resistin levels has also been investigated. Adiponectin is a metabolically active adipokine [73] that exerts protective effects against the development of insulin resistance, inflammation, and atherosclerosis [73,75]. In contrast, resistin was shown to induce adhesion molecules [76] and foam cell formation in experimental studies [77], and has been reported to play a role in obesity-associated subclinical inflammation, atherosclerosis, and CVD [78]. In contrast to rodents in which resistin is nearly exclusively derived from adipose tissue [79], in humans, resistin is abundantly expressed in inflammatory cells, particularly macrophages, which are important in inflammation and atherosclerosis [80]. In our previous study [73], serum adiponectin levels were negatively correlated with vascular inflammation represented by the mean TBR, while resistin levels were positively correlated with the same variable. In this study, vascular inflammation was independently associated with resistin levels even after adjusting for other cardiovascular risk factors, such as hsCRP [73].

Additionally, pericardial adipose tissue and visceral fat area were shown to be associated with vascular inflammation measured by ^18^F-FDG PET in 93 men and women without diabetes or CVD [81]. Pericardial adipose tissue and visceral fat area were also positively associated with major cardiovascular risk factors, including systolic blood pressure, LDL cholesterol, triglycerides, glucose, insulin resistance represented by HOMA-IR, and hsCRP, while they were inversely associated with HDL cholesterol [81].

## 7. Improvement in Vascular Inflammation Assessed by Positron Emission Tomography (PET) by the Medical Manage and Lifestyle Intervention Including Diet Control

Vascular inflammation may denote a dynamic aspect of atherosclerotic lesions, which varies during the course of atherosclerosis [82]. ^18^F-FDG uptake by atherosclerotic plaques is considered a transient phenomenon that is attenuated according to decreases in active inflammatory components [56]. Clinical and animal studies have explored whether vascular inflammation visualized by PET/CT can be reversed by interventions, including lifestyle modification and pharmacological therapy [56,60,83,84]. The antioxidant probucol was shown to decrease macrophage-rich fatty-streak lesions of atherosclerosis in Watanabe rabbits [85]. Ogawa et al. [84] reported that probucol treatment significantly reduced macrophage infiltration and ^18^F-FDG uptake by aortas, whereas intimal thickening was not altered in Watanabe heritable hyperlipidemic rabbits.

PPAR-gamma receptors are expressed by cells involved in the atherosclerotic process, including endothelial cells, smooth muscle cells, T-lymphocytes, and primarily macrophages [86,87]. In animal studies, thiazolidinediones, which are synthetic PPAR-gamma receptor ligands, were shown to have potent antiatherosclerotic properties suppressing the formation of atherosclerotic plaques, in addition to their antidiabetic effects [88,89]. Pioglitazone, a thiazolidinedione, was demonstrated to have vascular benefits [90,91] and preventive effects on all-cause mortality, myocardial infarction, and stroke in diabetic patients [92]. In this background, Vucic et al. [83] demonstrated that pioglitazone arrested the progression of vascular inflammation evaluated by ^18^F-FDG PET/CT in atherosclerotic rabbits. In the same study, immunoreactivity for macrophages and oxidized phospholipids from the aortas showed a significantly decreased value in the pioglitazone group compared to that in control rabbits [83].

Tahara et al. [60] reported that simvastatin therapy for three months reduced plaque inflammation as represented by decreased ^18^F^-^FDG uptakes in FDG-PET in humans. In the simvastatin group, the reduction in ^18^F^-^FDG accumulation was well correlated with the increase in HDL cholesterol but not with the decrease in LDL cholesterol [60], suggesting that an LDL-cholesterol-independent mechanism may be involved in the anti-inflammatory action of simvastatin on atherosclerotic plaques [60]. 

When analyzed with in vivo PET/CT scan and ex vivo gamma counting of excised aorta in hypercholesterolemic mice deficient in LDL receptor and expressing only apolipoprotein B-100, ^18^F-FDG uptake in the aorta was significantly lowered by a cholesterol-lowering diet [93]. At the same time, this dietary intervention effectively reduced plaque burden and macrophage count within atherosclerotic lesions on aortic histopathology [93]. Notably in a clinical setting, Lee et al. [56] reported that atherogenic risk reduction through lifestyle intervention reversed vascular ^18^F-FDG uptake in PET/CT in 60 healthy adults, and the extent of reduction in the ^18^F-FDG index correlated well with elevations in plasma HDL cholesterol levels [56]. Lifestyle modification strategy in this study [56] included individual dietary counseling provided by a registered dietitian. These results indicate that FDG-PET can also be used to track the effect of diet management, which is an important factor affecting lipoprotein oxidation and endothelial function, on plaque inflammation.

The results of previous studies suggest the utility of ^18^F-FDG PET for monitoring the effects of antiatherosclerotic therapies, including dietary intervention on vascular inflammation. Serial ^18^F-FDG PET may be a noninvasive tool for tracking the effects of therapeutic interventions on plaque inflammation and developing novel drugs that can recede inflammation on vulnerable plaques [56,60,83,84].

## 8. Conclusions

An increasing body of evidence supports that vascular inflammation, which is stimulated by modified LDLs and apoB-containing particles, and their interplay with macrophages plays a key action in atherosclerosis. ^18^F-FDG PET has emerged as a reliable imaging technique capable of identifying vascular inflammation associated with macrophage infiltration. Clinical studies suggest the utility of ^18^F-FDG PET for monitoring effects of dietary interventions and other antiatherosclerotic therapies on plaque inflammation. Although experimental and clinical studies provide indirect evidences that vascular inflammation may be affected by diets, further clinical studies addressing the effect of diet directly on atherosclerotic vascular inflammation and plaque vulnerability are needed. ^18^F-FDG PET can be a useful tool in identifying this issue.
